# Finding driver pathways in cancer: models and algorithms

**DOI:** 10.1186/1748-7188-7-23

**Published:** 2012-09-06

**Authors:** Fabio Vandin, Eli Upfal, Benjamin J Raphael

**Affiliations:** 1 Department of Computer Science, and Center for Computational Molecular Biology Brown University, 115 Waterman St., 4th Flr, Providence, RI 02912, USA

**Keywords:** Cancer, Somatic Mutations, Driver mutations, Pathways, Background mutation rate, Generative models

## Abstract

**Background:**

Cancer sequencing projects are now measuring somatic mutations in large numbers of cancer genomes. A key challenge in interpreting these data is to distinguish *driver mutations*, mutations important for cancer development, from *passenger* mutations that have accumulated in somatic cells but without functional consequences. A common approach to identify genes harboring driver mutations is a *single gene test* that identifies individual genes that are recurrently mutated in a significant number of cancer genomes. However, the power of this test is reduced by: (1) the necessity of estimating the *background mutation rate* (BMR) for each gene; (2) the mutational heterogeneity in most cancers meaning that groups of genes (e.g. pathways), rather than single genes, are the primary target of mutations.

**Results:**

We investigate the problem of discovering *driver pathways*, groups of genes containing driver mutations, directly from cancer mutation data and without prior knowledge of pathways or other interactions between genes. We introduce two generative models of somatic mutations in cancer and study the algorithmic complexity of discovering driver pathways in both models. We show that a single gene test for driver genes is highly sensitive to the estimate of the BMR. In contrast, we show that an algorithmic approach that maximizes a straightforward measure of the mutational properties of a driver pathway successfully discovers these groups of genes without an estimate of the BMR. Moreover, this approach is also successful in the case when the observed frequencies of passenger and driver mutations are indistinguishable, a situation where single gene tests fail.

**Conclusions:**

Accurate estimation of the BMR is a challenging task. Thus, methods that do not require an estimate of the BMR, such as the ones we provide here, can give increased power for the discovery of driver genes.

## Background

Cancer is a disease driven in part by somatic mutations that accumulate during the lifetime of an individual. These mutations include single nucleotide substitutions, small indels, and larger copy number aberrations and structural aberrations. A key challenge in cancer genomics is to distinguish *driver mutations*, mutations important for cancer development, from random *passenger mutations* that have accumulated in somatic cells but do not have functional consequences. Recent advances in DNA sequencing technologies allow the measurement of somatic mutations in large numbers of cancer genomes. Thus, a common approach to identify driver mutations, and the driver genes in which they reside, is to identify genes with recurrent mutations in a large cohort of cancer patients. The standard technique to identify such recurrently mutated genes is to perform a *single gene test*, in which individual genes are tested to determine if their observed frequency of mutation is significantly higher than expected [1-3]. This approach has identified a number of important cancer genes, but has not revealed all of the driver mutations and driver genes in individual cancers.

There are two difficulties with the identification of driver genes by a single gene test of recurrent mutation. First, the test requires a reasonable estimate of the *background mutation rate* (BMR) for each gene, or the rate at which passenger mutations occur in the gene. Obtaining such an estimate is not a straightforward task, as the BMR is not just the rate of somatic mutation per nucleotide per cell generation, but also must account for selection and clonal amplification in the somatic evolution of a tumor [1,4]. Second, it is widely observed that there is extensive mutational heterogeneity in cancer, with mutations occurring in different genes in different patients. This mutational heterogeneity is a consequence of both the presence of passenger mutations in each cancer genome, and the fact that driver mutations typically target genes in cellular signaling and regulatory pathways [5,6]. Since each of these pathways contains multiple genes, there are numerous combinations of driver mutations that can perturb a pathway important for cancer. This mutational heterogeneity inflates the number of patients required to distinguish passenger from driver mutations, as rare driver mutations may not be observed at frequencies above the background. An alternative to single gene tests is to test the recurrence of mutations in groups of genes derived from known pathways [7,8] or genome-scale gene interaction networks [9,10]. However, these approaches require prior knowledge of the interactions between genes/proteins, and this knowledge is presently far from complete. Moreover, pathway/network based approaches typically also require an estimate of the BMR.

The availability of somatic mutation data from increasing numbers of cancer patients motivates the question of whether it is possible to identify *driver pathways*, groups of genes with recurrent driver mutations, *de novo*; i.e. without prior knowledge of interactions between genes/proteins. At first glance, this seems implausible because there are an enormous number of possible sets of genes to consider. For example, there are more than 10^25^ sets of 7 human genes. However, we previously showed that mild additional constraints on the expected patterns of somatic mutations considerably reduce the number of gene sets to examine, and make *de novo* discovery of driver pathways possible [11]. These constraints are consistent with the current understanding of the somatic mutational process of cancer [6,12]. In particular, we assume that an important cancer pathway should be perturbed in a large number of patients. Thus, given genome-wide measurements of somatic mutations, we expect that a driver pathway will have high *coverage*: i.e. most patients will have a mutation in some gene in the pathway. Second, a driver mutation in a single gene of the pathway is often assumed to be sufficient to perturb the pathway. Combined with the fact that driver mutations are relatively rare, most patients exhibit only a single driver mutation in a pathway. Thus, we expect that the genes in a pathway exhibit a pattern of *mutually exclusive* driver mutations, where driver mutations are observed in exactly one gene in the pathway in each patient [13].

We emphasize that our assumption of mutual exclusivity holds only for driver mutations in the *same* pathway. It is well known that cancer genomes harbor driver mutations in multiple pathways, and the exclusivity assumption does not preclude the presence of such co-occurring, and possibly cooperative, driver mutations, examples of which are known [14,15]. Indeed, current estimates of the number of driver mutations and number of mutated pathways in a cancer genome are remarkably similar (≈10–15 [16,17]) suggesting that the assumption of approximately one driver mutation per pathway is not too strong of an assumption. It is also possible that multiple driver mutations are necessary to perturb a pathway and thus these mutations co-occur in patients. In this situation, there remains a large subset of genes in the pathway whose mutations are exclusive, e.g. a subset obtained by removing one gene from each co-occurring pair. The identification of these subsets of genes can be used as a starting point to later identify the other genes with co-occurring mutations.

### Our contribution

This work proposes a mathematical framework to study the problem of *de novo* discovery of driver genes and pathways. We define two generative models of driver mutations in cancer, the D > P model and the D=P model, and study the algorithmic complexity of the discovery problem in each of the models, both analytically and in simulations. The two generative models differ in how conditioning on a genome being from a cancer patient affects the ratio between the driver and passenger mutation probabilities in that genome. While the difference is relatively small, it has a major implication on the practicality of the standard single gene test for identifying the driver genes. In the first model we prove a bound on the number of patients required to detect all driver genes with high probability using a single gene test, while in the second model it is not possible to identify the driver genes using such a test for *any* number of patients.

Next, we study a weight function on sets of genes that quantifies the coverage and exclusivity properties of a driver pathway. We introduced this function in [11], and showed that finding sets with high weight provides an alternative approach for identifying driver mutations. Here, we prove that for both generative models, when mutation data from enough patients is available, the weight function is monotone in the number of discovered driver genes and is maximized by the driver pathway. Based on this observation we prove that a simple greedy algorithm identifies the driver pathways with high probability. This improves the result in [11], where we showed that the discovery problem is NP-hard for arbitrary mutation data and that a greedy algorithm performs well under different conditions that did not arise from a generative model of the data. We also show that our earlier Markov Chain Monte Carlo (MCMC) approach for identifying the driver pathways rapidly converges to the driver pathway in both generative models, thus improving the convergence result of [11] that considered arbitrary mutation data. These results show that we can identify driver pathways *without* an estimate of the background mutation rate (BMR), giving a more reliable and robust solution for the problem.

We complement our analytical results with experiments on simulated and real cancer sequencing data. For the first D > P model, we compare the number of patients required to identify driver genes using the single gene test with the number required using the greedy algorithm that maximizes the weight function. We show that the number of patients is similar when a perfect estimate of the BMR is available, but that the greedy algorithm requires a smaller number of patients when the estimate of the BMR deviates from its real value. For the second D=P model, we empirically verify that the single gene test cannot identify the driver genes even when a huge number of patients are analyzed, while the greedy algorithm correctly identifies all the driver genes. Finally, we test the performance of the greedy algorithm on mutation data from recent cancer sequencing studies, and show that the greedy algorithm can be used to identify the set of maximum weight on these datasets, even if the data is not guaranteed to satisfy the assumptions of our models. Our analytical and experimental results help characterize the limitations of detecting driver genes and pathways under reasonable models of somatic mutation.

In the remainder of this paper we consider the case in which the mutation matrix contains only one driver pathway. However, our results can be generalized to the case of multiple disjoint driver pathways. In particular the following iterative procedure identifies all driver pathways using our algorithms: after identifying a driver pathway, remove its genes from the mutation matrix, and look for driver pathways in the reduced mutation matrix.

## Methods

### Stochastic models for somatic mutations in cancer

In this section we introduce two stochastic models for somatic mutations in cancer. In both models driver mutations occur in *sets* of genes, which we refer to as *driver pathways*. Passenger mutations occur randomly across all genes. We assume that mutations have been measured in *n* genes in a collection of *m* cancer patients, and represent the somatic mutations as a *m* × *n* binary mutation matrix *A*. The entry *A*_*ig*_ in row *i* and column *g* is equal to 1 if gene *g* is mutated in patient *i*, and it is 0 otherwise. Let  be the set of all columns (genes). In both models, we assume that the mutation matrix contains a *driver pathway*: a subset D⊆G of genes, with |D|=k, such that in each patient *exactly one* of the genes of  contains a driver mutation. Thus, a driver pathway  exhibits the properties of high *coverage* – every patient has a mutation in a gene in  – and *mutual exclusivity* – no patient has a driver mutation in more than one gene in . In both models, random *passenger* mutations occur at random in all genes, including genes in . The difference between the two models is in the relative mutation rates in driver and passenger genes.

Following the hypothesis that cancer is triggered by a mutation in a driver gene, the sample of cancer patients can be viewed as a subset of a larger initial population. The genome of each member of the initial population was subject to random mutations, where each gene was mutated independently, and our sample is the subset of the initial population with a driver mutation in a gene of .

The first stochastic model captures the above intuition by modeling the distribution of mutations in patients as independent with fixed probability *q*, conditioning on having a driver mutation. The mutation matrix *A* is generated by the following process: in each row (patient) we choose one gene d∈D uniformly at random to contain the driver mutation, and set the corresponding entry *A*_*id*_ to 1. All other entries at that row are set to 1 with probability *q* < 1 and to 0 otherwise, and all events are independent. We call the parameter *q* the *passenger mutation probability*, as it is the probability that a gene contains a passenger mutation. We emphasize that *q* is greater than the BMR, since it is the probability that a *gene* has a passenger mutation. For example, estimates of the BMR are typically ≈10^−5^ – 10^−6^, and since the length of most genes is around 10^4^, we have that *q*≈10^−1^ – 10^−2^. We denote this model as the D > P model.

A possible limitation of the D > P model is that it implies a conditional distribution in which driver genes have higher expected frequency of mutation than the passenger genes (thus the name D > P model) in a cohort of patients. In practice the driver pathway  could contain dozens of genes, and some of them may have rare driver mutations. Thus the expected frequency of mutation of some genes in  may be indistinguishable from the expected frequency of mutation of some passenger genes. To examine this situation we introduce a second model, which we call the D=P model, in which all genes in  are mutated with the same probability in the patients, regardless of whether they are driver or passenger genes. Of course, this is a “worst case” model, as any cancer cohort with a reasonable number of patients will have some driver genes mutated at appreciable frequency. Nevertheless, we study the D=P model to consider the limits of driver pathway identification. The mutation matrix *A* in the D=P model is generated by the following process: in row (patient) *i* an entry *A*_*id*_ is chosen uniformly at random for d∈D and is set to 1. All other entries $A_{id^{\prime }}$ for $d^{\prime }\in D$ are set to 1 with probability $r=\frac{\mathrm{qk}-1}{k-1}$, and all entries *A*_*ig*_, for g∈G∖D are set to 1 with probability *q*. All events are independent. We require $q\geq \frac{1}{k}$ so that *r* is a proper probability. Note that for any g∈G the probability that *g* is mutated is the same since for d∈D, $\frac{1}{k}+(1-\frac{1}{k})r=q$.

Note that both models differ from a simple *binomial* model, where each entry of *A* is mutated independently with a fixed probability. Since we condition on each patient having at least one mutation in , the entries of *A* corresponding to genes in  are not independent. In what follows, we let $\Gamma (g)=\left\{i:A_{\mathrm{ig}}=1\right\}$ denote the set of patients in which a gene *g* is mutated. Similarly, for a set *M* of genes, let *Γ*(*M*) denote the set of patients in which at least one of the genes in *M* is mutated: $\Gamma (M)=\cup_{g\in M}\Gamma (g)$.

## Results

### Finding recurrently mutated genes

The standard approach to identify the driver genes is to identify recurrently mutated genes, i.e. those genes whose observed frequency of mutations is significantly higher than the expected *passenger mutation probability*[1-3]. This approach assumes a prior knowledge or a good estimate of the passenger mutation probability, the parameter *q* in our models. In particular if gene g∈G is not in the driver pathway , then the number of patients in which *g* is mutated among a collection of *m* cancer patients is described by a binomial random variable *B*(*mq*) with success probability *q*. If we know the value of *q* for each gene g∈G we can compute the probability *p*_*g*_ = Pr*B*(*mq*) ≥ |*Γ*(*g*)|] of observing gene *g* mutated in at least |*Γ*(*g*)| patients assuming g∉D (i.e., *p*_*g*_is the *p*-value for *g*). This approach is combined with a multi-hypothesis test to identify a list  of genes, each mutated in significantly more patients than expected. The pseudocode for such a test is given in Algorithm 1RMG. In Algorithm 1RMG we use Bonferroni correction for multiple hypothesis testing, that is we include in  the genes for which $p_{g}\leq \frac{\alpha }{n}$, for a fixed value *α*; the Bonferroni correction guarantees that the probability of reporting in  any gene not in  is bounded by *α*. Other corrections, like Benjamini-Hochberg [18] to control the *False Discovery Rate*, are possible. The results of this section also apply to these other corrections.

### Algorithm 1 **RMG**

Pseudocode of the algorithm for finding recurrently mutated genes, based on a single-gene test.**Input:** An *m* × *n* mutation matrix *A*, a probability *q* that a gene contains a passenger mutation in a patient, a significance level *α*. **Output:** Set  of recurrently mutated genes. 

1 O←∅;

2 **for**g∈G**do**

3 $\Gamma (g)\leftarrow \{i:A_{\mathrm{ig}}=1\}$;

4 $p_{g}\leftarrow \text{Pr}[B(m,q)\geq |\Gamma (g)|]$;

5 **if**$p_{g}\leq \frac{\alpha }{n}$**then**O←O∪{g};

6 **return**;

We first analyze the D > P model of Section “Stochastic models for somatic mutations in cancer”. We start by showing that if *q* is known and the number of patients is sufficiently large, then Algorithm 1RMG outputs all the driver genes with high probability.

#### Theorem 1

Suppose an *m* × *n* mutation matrix *A* is generated by the D > P model with D=k, the family wise error rate of the test is $\alpha =\frac{1}{2n^{\varepsilon }}$ and Algorithm 1RMG outputs . If $m\geq \frac{2k^{2}(1+\varepsilon )}{{\left(1-q\right)}^{2}}\ln2n$ for a constant *ε* > 0, then $\text{Pr}[O\neq D]\leq \frac{1}{n^{\varepsilon }}$.

#### Proof

The *p*-value calculations and the Bonferroni correction in Algorithm 1RMG guarantee that the probability that any gene g∉D is included in the output set  is bounded by $\alpha =\frac{1}{2n^{\varepsilon }}$. It remains to prove that if $m\geq \frac{2k^{2}(1+\varepsilon )}{{\left(1-q\right)}^{2}}\ln2n$ the probability that any d∈D is not included in  is bounded by $\frac{1}{2n^{\varepsilon }}$.

Consider a gene d∈D. Let *X*_*i*_=1 if gene *d* is mutated in patient *i*, and *X*_*i*_=0 otherwise. Note that for *i*≠*j*, *X*_*i*_ and *X*_*j*_ are independent. Let *X* be the number of patients in which *d* is mutated. We have $X=\overset{m}{\underset{i=1}{\sum }}X_{i}$. To compute **E**[*X*_*i*_] we observe that a driver gene is mutated with probability 1 when it contains the driver mutation, and with probability *q* otherwise. Since the gene *d* containing the driver mutation is chosen uniformly at random among all the *k* genes in , we have $E[X_{i}]=\frac{1}{k}+\left(1-\frac{1}{k}\right)q$. Thus $E[X]=\overset{m}{\underset{i=1}{\sum }}E[X_{i}]=m(\frac{1}{k}+\left(1-\frac{1}{k}\right)q)>\mathrm{mq}$. Let $t=\frac{1}{k}\left(\frac{1-q}{2}\right)$. By the Chernoff-Hoeffding bound:

$$\begin{array}{lll} \text{Pr}[X\leq E[X]-\mathrm{tm}]= & \text{Pr}[X\leq mE[X_{i}]-\mathrm{tm}]\; & \;\\ \leq & e^{-\frac{2m^{2}t^{2}}{m}}\leq \frac{1}{2n^{1+\varepsilon }}.\; & \; \end{array}$$

Since |D|<n, by union bound we have:

$$\begin{array}{lll} \text{Pr}[\exists d\in D\;\text{mutated in} & \leq (E[X]-\mathrm{tm})\;\text{patients}]\; & \;\\ & \leq n\frac{1}{2n^{1+\varepsilon }}=\frac{1}{2n^{\varepsilon }}.\; & \; \end{array}$$

Thus with probability at least $1-\frac{1}{2n^{\varepsilon }}$ all genes in  are mutated in at least **E**[*X*]−*tm* patients. Let *B*(*m*,*q*) be a binomial random variable with parameters *m,q*. Using the Chernoff-Hoeffding bound we can upper bound the *p*-value *p*_*d*_ that Algorithm 1RMG derives for *d* ∈ *D*:

$$p_{d}\leq \text{Pr}[|B(m,q)-\mathrm{mq}|\geq \mathrm{tm}]\leq e^{-2\frac{t^{2}m^{2}}{m}}\leq \frac{1}{2n^{1+\varepsilon }}.$$

Thus, with probability at least $1-\frac{1}{2n^{\varepsilon }}$ for any d∈D the number of patients with a mutation in *d* is such that its *p*-value satisfies *p*_*d*_ < *α*/*n* and thus it is included in the output set .

Theorem 1 shows that in the D > P model an estimate of the passenger mutation probability *q* and a sufficient number of patients are enough to identify the driver genes. This is not the case in the D=P model. It is easy to see that in D=P model the expected number of rows in which a column *g* is mutated is the same for all g∈G, that is for all g∈G we have **E**[|*Γ*(*g*)|] = *qm*. In fact, the number |*Γ*(*d*)| of patients in which a gene d∈D is mutated and the number |*Γ*(*g*)| of patients in which gene g∉D is mutated are both binomial random variables *B*(*m*,*q*). We thus have the following. □

#### Fact 1

Under the D=P model, the probability distribution of |*Γ*(*d*)| for d∈D and |*Γ*(*g*)| for g∉D are the same. Thus Algorithm 1RMG cannot identify the genes in  for any number of patients *m*.

### Finding recurrently mutated driver pathways

In this section we analyze a method that identifies the set  of driver genes with no prior information on the passenger mutation probability *q*, and works for both the D > P and D=P models. The method relies on a weight function *W*(*M*), defined on sets of genes, first introduced in [11]. The measure *W * quantifies the extent to which a set simultaneously exhibits both: (i) high *coverage*: most patients have at least one mutation in the set; (ii) high *exclusivity*: nearly all patients have no more than one mutation in the set.

For a set of genes, *M*, we define the coverage overlap $\omega (M)=\underset{g\in M}{\sum }|\Gamma (g)|-|\Gamma (M)|$. Note that *ω*(*M*) ≥ 0, with equality if and only if the mutations in *M* are mutually exclusive. To account for both the coverage, *Γ*(*M*), and the coverage overlap, *ω*(*M*), we define the weight function of *M*:

$$W(M)=|\Gamma (M)|-\omega (M)=2|\Gamma (M)|-\underset{g\in M}{\sum }|\Gamma (g)|.$$

Finding a set *M* of genes with maximum weight is in general a computationally challenging problem (it is NP-hard in the worst case [11]). Nonetheless, we showed in [11] that under some assumptions on the distribution of mutations in patients, a greedy algorithm will identify the maximum weight set. We also proposed a Markov Chain Monte Carlo (MCMC) approach that samples sets of genes with probability proportional to their weight.

Based on the coverage and exclusivity properties of a driver pathway we expect it has the highest weight among all sets of size *k*. In this section we formalize this intuition for our generative models and show that under the two models the maximum weight set is easy to compute. We use $M_{k}^{*}$ to denote the set of size *k* with maximum weight ( $M_{k}^{*}$ may not be unique).

We start with the D > P model. Note that the parameter *q* controls the expected number of passenger mutations in a set of *k* passenger genes. Since passenger mutations are relatively rare and *k* (the number of genes in a driver pathway) is relatively small, we expect that a set of *k* passenger genes will not have a mutation in the majority of the patients. Thus we assume that the probability 1−(1−*q*)^*k*^ that a set of *k* passenger genes contains at least one mutation in a patient is less than a constant $a<\frac{1}{2}$. Since 1−(1−*q*)^*k*^ ≈ *qk* we have $q\leq \frac{a}{k}$. For ease of exposition in what follows we set $a=\frac{1}{4}$, so that $q\leq \frac{1}{4k}$.

Let $M_{k,\ell }\subset G$ be a set of *k* genes with exactly *ℓ* genes of , that is $M_{k,\ell }=\{d_{1},d_{2},\ldots ,d_{\ell }\}\cup \{g_{1},\ldots ,g_{k-\ell }\}$ with $d_{j}\in D$ for 1 ≤ *j* ≤ *ℓ*, and $g_{j}\in G\setminus D$ for 1 ≤ *j* ≤ *k*−*ℓ*. We first prove that **E**[*W*(*Mk*,_*ℓ*_)] is monotone in *ℓ*.

#### Lemma 1

Let $q\leq \frac{1}{4k}$. For 0 ≤ *ℓ* ≤ *k*−1: $E[W(M_{k,\ell +1})]\geq E[W(M_{k,\ell })]+\frac{m}{2k}$.

#### Proof

Let *M* be any subset of , and let $E[W(M)]=\overset{m}{\underset{i=1}{\sum }}E[T_{i}]$, where *T*_*i*_ is the “contribution” of patient *i* to *W*(*M*), i.e. *T*_*i*_ = 2−*ℓ* if *ℓ* > 0 genes of *M* are mutated in *i*, and 0 otherwise. Note that *T*_*i*_ is the difference of two (dependent) random variables: *T*_*i*_ = *Y*_*i*_−*Z*_*i*_, where: *Y*_*i*_ = 0 if no gene of *M* is mutated in *i*, and 2 otherwise; *Z*_*i*_=*ℓ* if *ℓ* ≥ 0 genes of *M* are mutated in *i*.

Now consider *Mk*,_*ℓ*_ that contains a subset *L* of *ℓ* elements of . Consider the event *E*_*i*_ = “one of the genes of *L* is driver in patient *i*”, and ${Ē}_{i}$ its complement. We have $E[T_{i}]=E[T_{i}|E_{i}]\mathrm{Pr}[E_{i}]+E[T_{i}|{Ē}_{i}]\text{Pr}[{Ē}_{i}]$. For *Mk*,_*ℓ*_, when *E*_*i*_ holds, we have that *Y*_*i*_ = 2 (because one gene of *L* is mutated) and *Z*_*i*_ = 1 + *B*(*k*−1,*q*), where *B*(*k*,*q*) is a binomial random variable with parameters *k,q*. When *E*_*i*_does not hold, we have that *Y*_*i*_ = 2 with probability 1−(1−*q*)^*k*^ and *Z*_*i*_ = *B*(*k*,*q*) (since each gene of *Mk*,_*ℓ*_ is mutated independently with probability *q*). Thus for *Mk*,_*ℓ*_ we have

$$\begin{array}{lll} E[T_{i}]= & E[T_{i}|E_{i}]\mathrm{Pr}[E_{i}]+E[T_{i}|{Ē}_{i}]\text{Pr}[{Ē}_{i}]\; & \;\\ = & (2-(1+q(k-1)))\frac{\ell }{k}+(2(1-{\left(1-q\right)}^{k})\; & \;\\ & -\mathrm{qk})\left(1-\frac{\ell }{k}\right).\; & \; \end{array}$$

Thus $E[M_{k,\ell }]=m((2-(1+q(k-1)))\frac{\ell }{k}+(2(1-{\left(1-q\right)}^{k})-\mathrm{qk}))\left(1-\frac{\ell }{k}\right)$.

Analogously for *Mk*,_*ℓ* + 1_we have $E[T_{i}]=(2-(1+q(k-1)))\frac{\ell +1}{k}+(1-\frac{\ell +1}{k})(2(1-{\left(1-q\right)}^{k})-\mathrm{qk})$ and $E[M_{k,\ell +1}]=m((2-(1+q(k-1)))\frac{\ell +1}{k}+(2(1-{\left(1-q\right)}^{k})-\mathrm{qk}))(1-\frac{\ell +1}{k})$.

Thus we have:

$$\begin{array}{lll} \; & E[W(M_{k,\ell +1})]-E[W(M_{k,\ell })]\; & \;\\ & \;\;=m\left(\frac{2-(1+q(k-1))}{k}-\frac{2(1-{\left(1-q\right)}^{k})-\mathrm{qk}}{k}\right)\; & \;\\ & \;\;=m\left(\;-\frac{1}{k}+\frac{q}{k}+\frac{2}{k}{\left(1\;-\;q\right)}^{k}\right)\; & \;\\ & \;\;\geq m\left(-\frac{1}{k}+\frac{q}{k}+\frac{2}{k}-2q\right)\; & \;\\ & \;\;=m\left(\frac{1}{k}+\frac{q}{k}-2q\right)\; & \;\\ & \;\;\geq m\frac{1}{2k}.\; & \; \end{array}$$

where the first inequality follows from (1−*q*)^*k*^ ≥ 1 −*qk*, and the last inequality follows from $q\leq \frac{1}{4k}$ and *q* > 0.

Next we show that for sufficiently large number of patients *m*, the random value *W*(*Mk*,_*ℓ*_) is concentrated near its expectation. □

#### Theorem 2

Suppose an *m* × *n* mutation matrix *A* is generated by the D > P model with |D|=k and $q\leq \frac{1}{4k}$. For $m\geq 8k^{3}(k+\varepsilon )\ln n$, and for 0 ≤ *ℓ* ≤ *k*−1, $\mathrm{Pr}[\exists M_{k,\ell }\;\text{s.t.}\;|W(M_{k,\ell })-E[W(M_{k,\ell })|\geq \frac{m}{4k}]\leq \frac{1}{n^{\varepsilon }}.$

#### Proof

Let *M* = {*g*_1_*g*_2_,…,*g*_*k*_} be a set of *k* genes. Consider the sequence  of random variables *X*_1,1_*X*_1,2_,…,*X*1,_*k*_*X*_2,1_*X*_2,2_,…,*X*2,_*k*_,…,*Xm*,_*k*_, with *Xi*,_*j*_ = 1 if gene *g*_*j*_ is mutated in patient *i*, and *Xi*,_*j*_ = 0 otherwise. (Note that *Xi*,_*j*_ are not mutually independent since at least one gene in the driver pathway  is mutated in each patient.) The random variable *W*(*M*) is determined by the sequence . Now consider the *Doob martingale* (see [19]) *Zi*,_*j*_ = **E***W*(*M*)|*X*_1,1_,…,*Xi*,_*j*_, 0 ≤ *i* ≤ *m*, 1 ≤ *j* ≤ *k*. Note that *Zm*,_*k*_ = *W*(*M*), and **E***Zm*,_*k*_ = **E***W*(*M*)]. Since changing the value of any of the random variables in  changes *W*(*M*) by at most 1 and there are *km* such random variables, by Azuma-Hoeffding inequality we have that, for all *t* > 0:

$$\text{Pr}[|W(M)-E[W(M)]|\geq t]\leq 2e^{-\frac{2t^{2}}{\mathrm{km}}}.$$

Setting *t* = *m*/(4*k*), and summing over all $\left(\begin{array}{c} n\\ k \end{array}\right)$ possible choices of the set *M* gives the result.

Combining the results of Lemma 1 and Theorem 2 we have □

#### Corollary 1

If $m\geq 8k^{3}(k+\varepsilon )\ln n$, then $\text{Pr}[M_{k}^{*}\neq D]\leq \frac{1}{n^{\varepsilon }}$.

Corollary 1 shows that with sufficient number of patients the set  can be identified by finding the set of maximum weight, without an estimate of the passenger mutation probability *q* We previously showed in [11] that with an arbitrary mutation distribution identifying the set of maximum weight is NP-Hard. However, a corollary of Theorem 2 shows that in the D > P model computing a set of maximum weight is easy.

#### Corollary 2

If $m\geq 8k^{3}(k+\varepsilon )\ln n$ and $q\leq \frac{1}{4k}$, Algorithm 2 GreedyWeight that computes the weight function of up to *O*(*nk*) sets finds $M_{k}^{*}$ with failure probability $\leq \frac{1}{n^{\varepsilon }}$.

#### Proof

The pseudocode for Algorithm 2 GreedyWeight is given below. Theorem 2 guarantees that if ^*g*∗^ is inserted in *M*, it is in , and that when a gene g∈M∖D is considered, it will be switched with a gene $g^{\prime }\in D\setminus M$. □

### Algorithm 2 **GreedyWeight**

Pseudocode of the greedy algorithm for finding the set *M* of maximum weight *W*(*M*). **Input:** An *m* × *n* mutation matrix *A*, integer *k* > 0.**Output:** Set ^*M*∗^ of maximum weight *W*(^*M*∗^). 

1 M ←k random columns from *A*;

2 $M^{*}\leftarrow M$;

3 **for***g*∈*M***do**

4 $g^{*}\leftarrow {\text{arg max}}_{g^{\prime }\in G\setminus M^{*}}\{W(M^{*}\setminus \{g\}\cup \{g^{\prime }\})\}$;

5 **if**$W(M^{*}\setminus \{g\}\cup \{g^{*}\})>W(M^{*})$**then**$M^{*}\leftarrow M^{*}\setminus \{g\}\cup \{g^{\prime }\}$;

6 **return**^*M*∗^;

We now consider the D=P model. Analogously to what we proved under the D > P model, we prove that maximizing the weight function *W * identifies the driver pathway  when mutation data from enough patients is available.

#### Theorem 3

Suppose an *m* × *n* mutation matrix *A* is generated by the D=P model with |D|=k. If $m\geq \frac{k^{3}(k+\varepsilon )}{2{\left(1-q\right)}^{2k+2}}{\left(\frac{k-1}{k}\right)}^{2k}\ln n$, then $\text{Pr}[M_{k}^{*}\neq D]\leq \frac{1}{n^{\varepsilon }}$.

#### Proof

Consider *ℓ* ≥ 1. As in the proof of Lemma 1, we have that for any pair of sets *Mk*,_*ℓ*_,*M*_*k*,*ℓ*  +  1_of size *k* containing *ℓ* and *ℓ* + 1 elements of  respectively, we have: $E[W(M_{k,\ell +1}]\geq E[W(M_{k,\ell })]+m\frac{2}{k}$${\left(1\;-\;q\right)}^{k+1}{\left(\frac{k}{k-1}\right)}^{k}$. Using the Azuma-Hoeffding inequality with $t\;=\;m\frac{1}{k}{\left(1\;-\;q\right)}^{k+1}{\left(\frac{k}{k-1}\right)}^{k}$ we have that $\mathrm{Pr}[\exists M_{k,\ell }\;\text{s.t.}\;|W(M_{k,\ell })\;-\;E[W(M_{k,\ell })|\;\geq \;t]\leq \frac{1}{n^{\varepsilon }}$ for all *Mk*,_*ℓ*_. The theorem follows combining these two properties.

We prove that a simple greedy algorithm, similar to Algorithm 2 GreedyWeight that we proposed for the D > P model, identifies the set $M_{k}^{*}$ of maximum weight under the D=P model. □

#### Corollary 3

If $m\geq \frac{k^{3}(k+\varepsilon )}{2{\left(1-q\right)}^{2k+2}}{\left(\frac{k-1}{k}\right)}^{2k}\ln n$, a greedy algorithm that computes the weight function of up to *O*(*n*2) sets finds $M_{k}^{*}$ with failure probability $\leq \frac{1}{n^{\varepsilon }}$.

#### Proof

Start with an arbitrary set *M* of *k* genes. By the proof of Theorem 3, if *M* already contains at least one gene of , Algorithm 2 GreedyWeight produces the set  in output with failure probability $\leq \frac{1}{n^{\varepsilon }}$. Thus we only need to make sure that the initial set *M* includes at least one gene of . To do this, take an arbitrary pair *g*_1_,*g*_2_ of genes in *M*, and find the pair $(g_{3},g_{4})\in G\setminus M$ that maximizes $W(M\setminus \{g_{1},g_{2}\}\cup \{g_{3},g_{4}\})$. Then exchange *g*_1_,*g*_2_for _*g*3_,*g*_4_ if $W(M\setminus \{g_{1},g_{2}\}\cup \{g_{3},g_{4}\})>W(M)$. Running Algorithm 2 GreedyWeight from the resulting set *M* gives the result.

Thus under the D=P model we identify the driver pathway  by maximizing *W*(*M*). Recall that Algorithm 1RMG cannot find driver genes under this model (Section “Finding recurrently mutated genes”, Fact 1). Also note that when *q* ≤ 1/2 and the probability (1−*q*)^*k*^ that a set of *k* genes in G∖D is not mutated in a patient is greater than $\frac{1}{2}{\left(\frac{k-1}{k}\right)}^{k}$ (this occurs when passenger mutations are relatively rare, for example when *q* ≈ 1/*k*) the bound on *m* in Corollary 3 is the same as the bound in Corollary 2. That is, the weight *W * identifies the set  under both the D > P and D=P models with the same number of patients.

For completeness, we also analyze the Monte-Carlo Markov Chain approach proposed in [11] to sample sets of genes with distribution exponentially proportional to their weight. The pseudocode for the sampling procedure used by the Monte-Carlo Markov Chain approach is given in Algorithm 3 MCMC-Sampling. It is easy to verify that the chain is ergodic with a unique stationary distribution $\Pi (M)=\frac{e^{\mathrm{cW}(M)}}{\underset{R\in {ℳ}_{k}}{\sum }e^{\mathrm{cW}(R)}}$, where ${ℳ}_{k}=\{M\subset G||M|=k\}$. The efficiency of this algorithm depends on the speed of convergence of the Markov chain to its stationary distribution. □

### Algorithm 3 **MCMC-Sampling**

Pseudocode of the sampling procedure for the MCMC algorithm.**Input:** Current state ^*M*(*t*)^**Output:** Next state ^*M*(*t* + 1)^

1 w← gene chosen uniformly at random from ;

2 v← gene chosen uniformly at random from ^*M*(*t*)^;

3 $P(M^{\left(t\right)},w,v)\leftarrow \text{min}[1,e^{\mathrm{cW}(M^{\left(t\right)}\setminus \{v\}\cup \{w\})-\mathrm{cW}(M^{\left(t\right)}\;)}]$;

4 With probability *P*(^*M*(*t*)^,*w*,*v*) set $M^{\left(t+1\right)}\leftarrow M^{\left(t\right)}\setminus \{v\}\cup \{w\}$, otherwise $M^{\left(t+1\right)}\leftarrow M^{\left(t\right)}$;

In [11], we show that there is a non-trivial interval of values for *c* for which the chain is rapidly mixing without assuming any generative model for the mutation matrix. Applying the analysis in [11] to the D > P and D=P models requires $0<c<\frac{1}{k}$. However, applying Lemma 1 and 2 under the D > P model, and Theorem 3 under the D=P model we show that for any *c* > 0 the process rapidly converges to the set .

#### Theorem 4

Suppose an *m* × *n* mutation matrix *A* with |D|=k is generated by the D > P model with $q\leq \frac{1}{4k}$, or the D=P model with $q\leq \frac{1}{2}$ and ${\left(1-q\right)}^{k}\geq \frac{1}{2}{\left(\frac{k-1}{k}\right)}^{k}$. For $m\geq 8k^{3}(k+\varepsilon )\ln n$ and any *c* > 0, the MCMC converges to the set > in *O*(*nk*log*k*) iterations with probability $\geq 1-\frac{1}{n^{\varepsilon }}$.

#### Proof

As stated above, the analysis of [11] applied to the D > P and D=P models gives the result for $0<c<\frac{1}{k}$. We now prove that the result holds for $c\geq \frac{1}{k}$. The theorem follows by combining the two cases.

Consider the MCMC and assume there is no time step *t* such that the chain transitions from a set *Mk*,_*ℓ* + 1_containing *ℓ* + 1 genes in  to a set *Mk*,_*ℓ*_containing *ℓ*genes in . Note that if the MCMC is in a state containing *ℓ* < *k*genes of , it will transition to a state with *ℓ* + 1 genes of  (that is, *w*∈*D*and v∉D are chosen) with probability ≥1/(*kn*). From a coupon collector analysis we have that, if the MCMC never transitions from a set *M*_*ℓ* + 1_ containing *ℓ* + 1 genes in  to a set *M*_*ℓ*_ containing *ℓ* genes in , the MCMC converges to the set  after 2knln(2kn) steps with probability at least 1−(2*n*)^−1^.

We now bound the probability that the MCMC moves from a set *M*_*ℓ* + 1_to a set *M*_*ℓ*_in the 2knln(2kn) steps before reaching state . Given the choice of *m*, from Theorem 2 and Theorem 3 we have that the probability that in a particular step the MCMC moves from a set *M*_*ℓ* + 1_ to a set *M*_*ℓ*_is bounded by $e^{-c\frac{m}{k}}$. The theorem follows by union bound on the 2knln(2kn) steps and from the bounds of *m* and *c*. □

### Experimental results: simulated data

In this section we compare the single gene test (Algorithm 1RMG) with the driver pathway approach (using the weight function *W*(*M*)) to detect the set of driver genes using mutation data simulated using the D > P and the D=P model. In particular, we use Algorithm 2 GreedyWeight of Section “Finding recurrently mutated driver pathways” to identify the set $M_{k}^{*}$ of maximum weight, where k=|D|.

We first consider the D > P model, generating mutation data with k=|D|=20, *n* = 10000, and for different values of *q*. In particular we considered *q*∈{0.0125;0.0075;0.001}. We set *α* = 0.005 for Algorithm 1RMG which corresponds to *ε* = 0.5. To compare the performance of the two algorithms, we measured the minimum number of patients required to detect the driver pathway  over a range of estimates of the passenger mutation probability *q*. Specifically, let *E*_*s*(*q*)_ = “estimate *s*(*q*) of *q* is used by Algorithm 1RMG”. Let $m_{R,x}(s(q))={\text{min}}_{m}\{\text{Pr}[O=D|E_{s(q)}]>x\}$ be the minimum number of patients required for Algorithm 1RMG to output O=D with probability  > *x* over all *m* × *n* mutation matrices generated by the model when the estimate *s*(*q*) is used. Similarly, let  be the output of Algorithm 2 GreedyWeight. Let $m_{G,x}={\text{min}}_{m}\{\text{Pr}[P=D]>x\}$ be the minimum number of patients required for Algorithm 2 GreedyWeight to output  with probability  > *x* over all *m* × *n* mutation matrices generated by the model. Recall that *m*_*G*,*x*_ does not depend on *s*(*q*) by Corollary 2.

**Figure 1:**
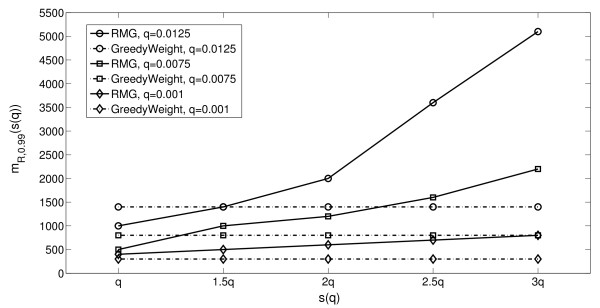
**Comparison of Algorithm 1**RMG** and Algorithm 2 **GreedyWeight**.** Comparison between the estimate of the number of patients *m*_*R*,0.99_(*s*(*q*)) required to identify the driver pathway  with Algorithm 1 RMG, for different estimates *s*(*q*) of the probability *q* and different values of *q*, and the number of patients _*mG*,0.99_required to identify  with Algorithm 2 GreedyWeight.

Figure 1 shows the values of *m*_*R*,0.99_(*s*(*q*)) and *m*_*G*,0.99_ as a function of *s*(*q*). We varied *s*(*q*) starting from *s*(*q*)=*q* (i.e., *q* is perfectly estimated) and gradually increased *s*(*q*) while maintaining *s*(*q*) < 1/*k*. The latter condition assures that *s*(*q*) is strictly smaller than the expected probability of mutation of any gene in , a necessary condition for Algorithm 1RMG to be able to identify . To compare *m*_*R*,0.99_ and *m*_*G*,0.99_ we generated 100 mutation matrices for each *m*_*i*_=*i*×100 patients for 1 ≤ *i* ≤ 52 and obtained an empirical estimate of *m*_*R*,0.99_ and _*mG*,0.99_.^a^ For a fixed *q*, Figure 1 shows that *m*_*R*,0.99_(*s*(*q*)) is monotonically increasing with *s*(*q*), and that as expected both *m*_*R*,0.99_(*s*(*q*)) and *m*_*G*,0.99_ decrease using lower values of *q*. For *q* = 0.0125 and *q*=0.0075, when the estimate of *q* is perfect Algorithm 2 GreedyWeight requires more patients than Algorithm 1RMG to correctly identify the set , but when the estimate *s*(*q*) is larger than the true value of *qm*_*R*,0.99_(*s*(*q*)) increases and becomes much larger than _*mG*,0.99_. (Typically, an overestimate of *q* is used so that the test for recurrent genes in conservative [20]). Note that even when *s*(*q*)=*qm*_*G*,0.99_ is close to *m*_*R*,0.99_(*q*), and that for *q*=0.001, _*mG*,0.99_ < *m*_*R*,0.99_(*s*(*q*)) even when *s*(*q*)=*q*, while the bounds in Theorem 1 and in Corollary 2 give $\frac{m_{G,0.99}}{m_{R,0.99}(q)}\geq 1000$ for all the parameters we used. Similar results were obtained when comparing _*mR*,0.95_(*s*(*q*)) and _*mG*,0.95_; i.e. the minimum number of patients for which Algorithm 1RMG and Algorithm 2 GreedyWeight report the driver set  at least 95% of the time(data not shown).

We also considered the case *s*(*q*) < *q* where the estimate of *q* is smaller than its true value. In this case, some genes not in  (false positives) are eventually reported by Algorithm 1RMG. For example, with *m* = 1000 patients and *q* = 0.0125, when *s*(*q*) = *q*, the correct set of genes (with no false positives) were reported. However, when *s*(*q*) = 0.8*q*Algorithm 1RMG reports false positives in approximately 16% of the datasets. In contrast, Algorithm 2 GreedyWeight does not suffer from this problem, since it does not require an estimate *s*(*q*) of *q*.

We now consider the D=P model, generating mutation data with k=|D|=20, *q* = 0.05 and *n* = 120. As stated in Fact 1, Algorithm 1RMG cannot identify the genes in  for any number *m* of patients; we checked this property for values of *m* up to 10^7^. For Algorithm 2 GreedyWeight we again estimated *m*_*G*,0.99_ as described above generating 100 mutation matrices for each *m*_*i*_ = *i*×1000 patients for 1 ≤ *i* ≤ 100, and obtained that *m* = 95000 patients suffices for GreedyWeight to correctly output exactly the genes in , while the bound of Corollary 3 gives that more than 4×10^5^patients are required for the parameters we used.

In the above experiments we provided the correct parameter *k* in input to the Algorithm 2 GreedyWeight. In practice, the exact value of *k* is not known. However, when the number *m* of patients satisfies the bound of Corollary 2 (resp., Corollary 3) in the D > P (resp., D=P) model, then the weight W(D) of the set  is greater than the weight of *any* other set of genes. We therefore implemented a modified version of the greedy algorithm that takes as input an upper bound $k_{\max}$ on the size of , runs Algorithm 2 GreedyWeight for all values *k* with $2\leq k\leq k_{\max}$ and outputs the set (of any size) of maximum weight found in the different runs. We repeated the experiments above for the D > P model with *n* = 10000 and *q* = 0.0125 and for the D=P model using $k_{\max}=22$ for this algorithm, and obtained the same estimates of _*mG*,0.99_reported above. This show that even when the exact value of *k* is not known, Algorithm 2 GreedyWeight can correctly identify .

### Experimental results: cancer sequencing data

Finally, we tested Algorithm 2 GreedyWeight on mutation data coming from three different cancer sequencing studies, as described in [11]. In particular we analyzed cancer mutation data from: lung adenocarcinoma [21], glioblastoma [3], and multiple cancer types [22]. The mutation matrices were prepared using the same procedure described in [11]. Since not all genes have been assayed for mutations in these studies, there is no guarantee that the assumptions of our models hold for these datasets. In addition, the number of mutated patients in the studies is small compared to the bounds our analytical and empirical results suggest for Algorithm 2 GreedyWeight to find the set of maximum weight. Nonetheless, for each of the three datasets we attempted to use Algorithm 2 GreedyWeight to find the set of maximum weight we reported in [11], using the parameter *k* given by the size of the sets found in [11].

Since the output of Algorithm 2 GreedyWeight depends on the choice of the initial random set (the set *M* on Line 1 of Algorithm 2), we run Algorithm 2 GreedyWeight 100 times (i.e., starting from 100 different random initial sets). For the mutation data from multiple cancer types, Algorithm 2 GreedyWeight *always* reports the set of maximum weight; for the mutation data from the gliblastoma study, the set of maximum weight is reported by Algorithm 2 GreedyWeight in 58% of the runs. For the lung adenocarcinoma mutation data, Algorithm 2 GreedyWeight reports the set of maximum weight in 43% of the runs, and no other set is reported more frequently. These results show that Algorithm 2 GreedyWeight can be used to identify genes in driver pathways on data from cancer sequencing studies containing a modest number of patients.

## Conclusions

We investigate the problem of detecting recurrently mutated genes and pathways using two simple generative models of driver mutations in cancer: the D > P model and the D=P model. In the D > P model, the driver mutation probability is larger than the passenger mutation probability. We prove a bound on the number of patients required to detect all driver genes with high probability using a single gene test of recurrence. In the D=P model, the driver mutation probability and passenger mutation probability cannot be distinguished, and thus it is impossible to identify driver genes using the single gene test for *any* number of patients. We prove that under either model, the weight function on sets of genes that we defined in [11] is maximized by a driver pathway. Thus, with mutation data from enough patients, it is possible to identify driver pathways *without* an estimate of the passenger mutation probability *q*. In particular, we show that a simple greedy algorithm finds driver pathways with high probability. We also show that an MCMC approach converges rapidly. We present results on simulated data showing that the greedy algorithm successfully identifies the driver pathway with fewer patients than the single gene test when the estimate of *q* deviates from its real value. Finally, we show that the greedy algorithm can find driver genes and driver pathways in real cancer sequencing data containing a modest number of patients.

In practice, any test that identifies driver genes by recurrent mutations requires a good estimate of the passenger mutation probability *q*. An underestimate of *q* leads to false positive predictions of driver genes, while an over estimate (i.e. a conservative estimate to minimize false positives) increases the number of patients required to find driver genes. The passenger mutation probability is derived from the background mutation rate (BMR), which is difficult to measure as it depends on a number of parameters whose values are not easily determined. There has been extensive discussion in the community about appropriate ways to estimate the BMR and find recurrently mutated genes [1,4]. Methods that do not require an estimate of the BMR, as the ones we provide here, can give increased power for the discovery of driver genes. However, further study of more sophisticated mutation models is necessary. For example, we assume a constant passenger mutation probability *q* across all genes, but models that allow *q* to vary by gene would be useful in applications and warrant further investigation.

## Consent

Written informed consent was obtained from the patient for publication of this report and any accompanying images.

## Endnotes

^a^ We use the empirical estimates of *m*_*R*,0.99_(*q*) and *m*_*G*,0.99_ only to compare the performance of Algorithm 1RMG and Algorithm 2 GreedyWeight, and to show how *m*_*R*,0.99_ and _*mG*,0.99_vary by changing the parameter *q*. Therefore we do not need extremely accurate estimates of of *m*_*R*,0.99_(*q*) and _*mG*,0.99_, that would require the generation of more mutation matrices and the inclusion of more values of *m*_*i*_.

## Competing interests

FV and BJR declare that they have no competing interests. EU has financial interest in Nabsys Inc.

## Authors’ contributions

All authors contributed to the analytical part of the work. FV wrote the software and ran the experiments. All authors read and approved the final manuscript.
